# Supplementary material for: Cost-effectiveness of Vitamin A supplementation among children in three sub-Saharan African countries: An individual-based simulation model using estimates from Global Burden of Disease 2019
Source: PLoS One. 2022 Apr 7;17(4):e0266495. doi: 10.1371/journal.pone.0266495 (PMC8989187; doi:10.1371/journal.pone.0266495)
Supplement: S1 Appendix — (DOCX) [file pone.0266495.s001.docx]

**S1 Appendix: Simulation Inputs**

In order to run a Vivarium simulation, various inputs are necessary to connect different parts of the model. With these inputs, we can project how changes in upstream components like coverage affect downstream components such as outcomes (see **Fig 1**). For many inputs, we utilize GBD estimates to model these relationships.

However, for some inputs that were either outdated or missing, we performed our own meta-analysis. These inputs were the relative risk of lack of VAS on VAD, and baseline coverage of VAS in Nigeria and Kenya.

To determine the relative risk of VAS on VAD, we used a combination of the search phrases “Vitamin A supplementation” AND “Vitamin A deficiency” AND “relative risk” in Google Scholar and ((Vitamin A supplementation) AND (Vitamin A deficiency)) AND (relative risk[Title/Abstract]) in PubMed to locate relevant articles. We received 1,020 hits from Google Scholar and 33 hits from PubMed (**S1 Fig**). The initial screen focused on literature that gathered primary data for children between 6-60 months. We found that a large fraction of the articles found the relative risk of lack of VAS on a cause of death. We screened out papers that did not specify a relative risk directly between VAS and VAD. Next, we determined which of these journal articles are eligible based on their definitions of VAS and VAD. We only used articles that designated the frequency of VAS as two doses per year or one dose every six months, and described VAD as a serum retinol concentration of less than 0.70 µmol/L [8].

**
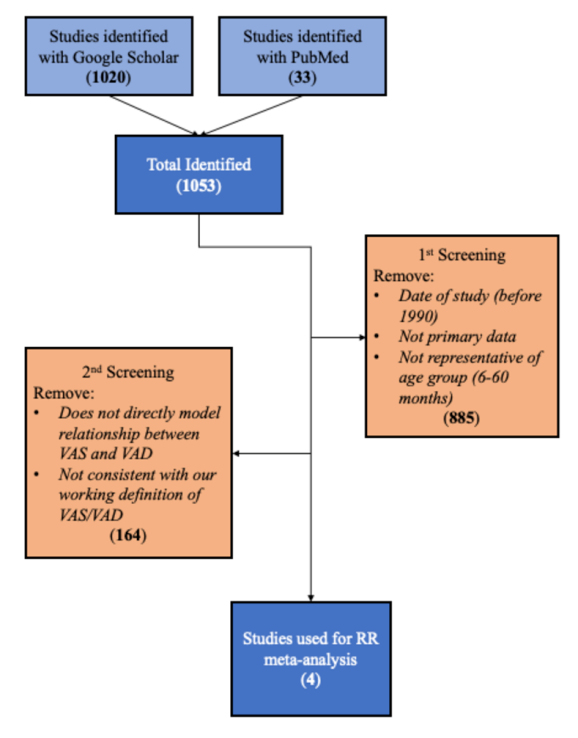
**

**S1 Fig: Relative Risk Literature Search Flow diagram**

Using these papers, we conducted a fixed-effect meta-analysis for the relative risk (**S2 Fig**). For each article, we extracted the sample size as well as number of individuals surveyed with/without supplementation and with/without VAD to determine the weights for each study.

**
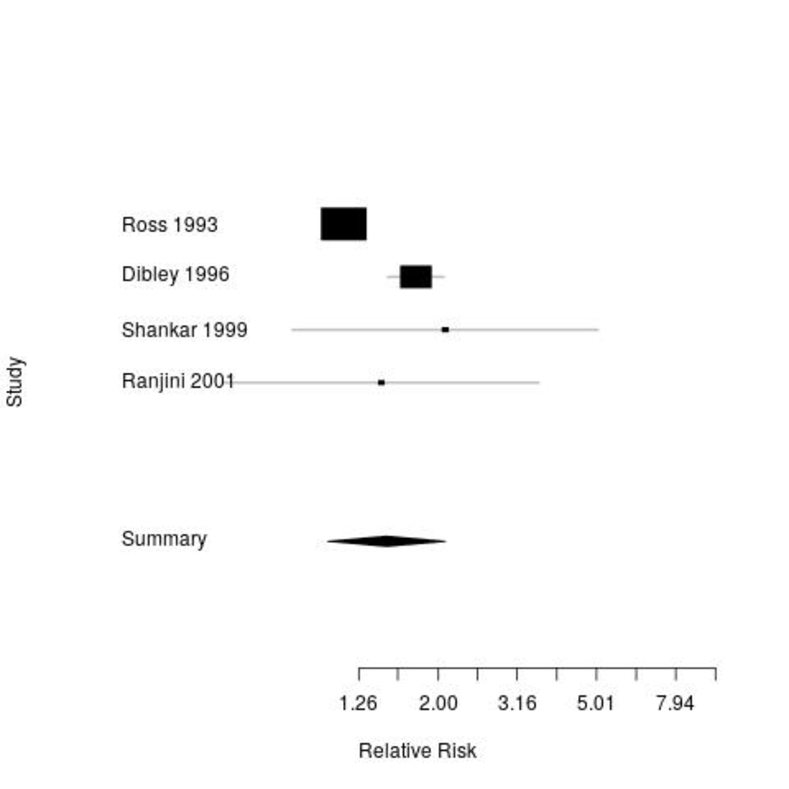
**

**S2 Fig: Literature Search results for Relative Risk of Lack of Vitamin A Supplementation on Vitamin A Deficiency**

To determine the baseline coverage of VAS in Nigeria and Kenya, we searched for publications in Google Scholar with the search term "Vitamin A Supplementation coverage" AND ("Kenya" OR "Nigeria") and in PubMed with the search term ((Kenya) OR (Nigeria)) AND (Vitamin A Supplementation coverage). In addition, we included DHS reports from these nations as part of our analysis. We screened journal articles and DHS surveys to only include those that were published after 2002 (15 years before the beginning of the simulation) and relevant to the age group. Finally, we selected which journal articles were eligible based on their definition of VAS, and if they are measuring campaign-based mechanisms for supplementation (**S3 Fig**).

**
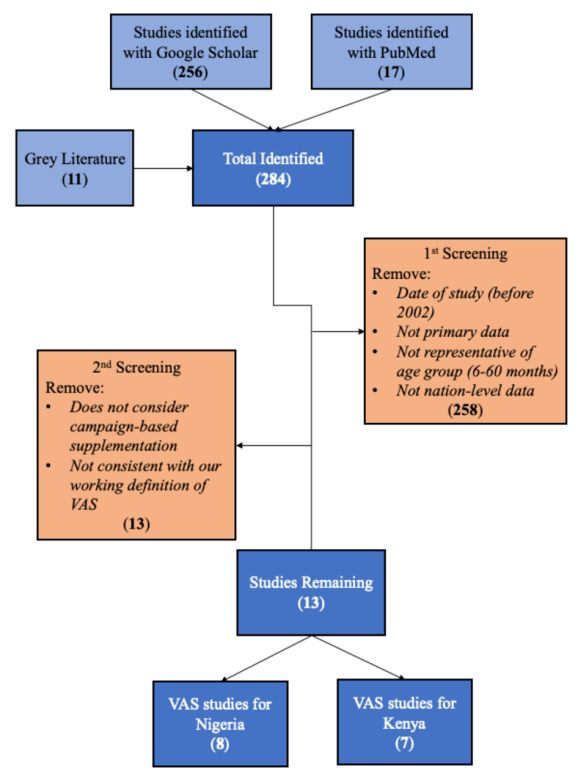
**

**S3 Fig: Baseline Coverage Literature Search Flow diagram**

We used the remaining sources for our analysis. Some studies are used for calculating the baseline coverage in both Nigeria and Kenya. We extracted sample size and the coverage from each article to calculate the weights for the fixed-effect meta-analysis (**S4 Fig, S5 Fig**).

**
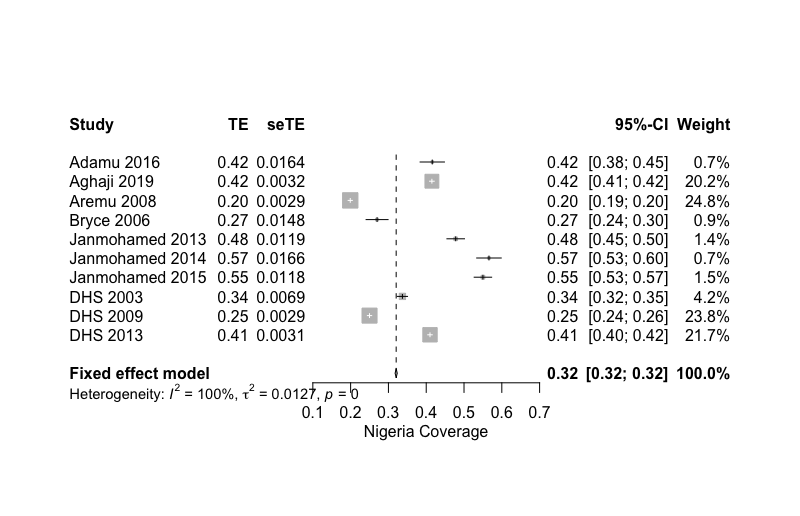
**

**S4 Fig: Literature Search results for Baseline Coverage of Vitamin A Supplementation in Nigeria**

**
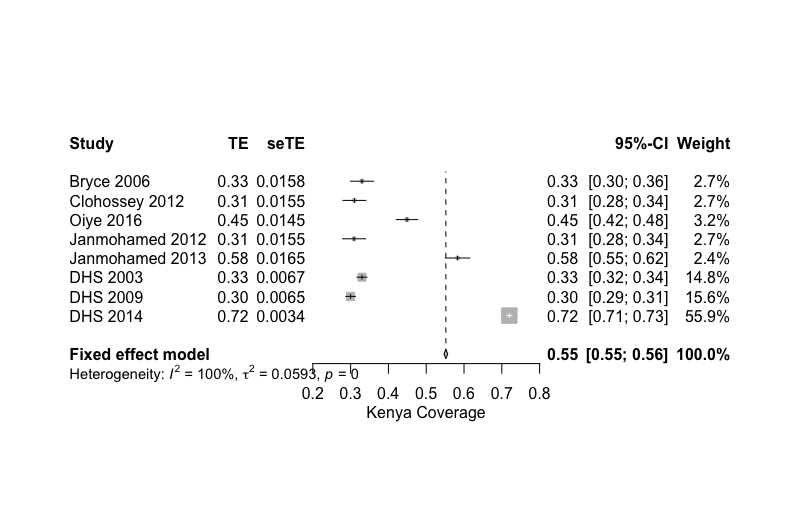
**

**S5 Fig: Literature Search results for Baseline Coverage of Vitamin A Supplementation in Kenya**
