# Supplementary material for: Cost-effectiveness of Vitamin A supplementation among children in three sub-Saharan African countries: An individual-based simulation model using estimates from Global Burden of Disease 2019
Source: PLoS One. 2022 Apr 7;17(4):e0266495. doi: 10.1371/journal.pone.0266495 (PMC8989187; doi:10.1371/journal.pone.0266495)
Supplement: S2 Appendix — (DOCX) [file pone.0266495.s002.docx]

**S2 Appendix: Yearly Results**

**S1 Table: Simulation Results for Vitamin A Supplementation scale-up costs, coverage, and outcomes by country and year**
