# Supplementary material for: Cost-effectiveness of Vitamin A supplementation among children in three sub-Saharan African countries: An individual-based simulation model using estimates from Global Burden of Disease 2019
Source: PLoS One. 2022 Apr 7;17(4):e0266495. doi: 10.1371/journal.pone.0266495 (PMC8989187; doi:10.1371/journal.pone.0266495)
Supplement: Attachment — Submitted filename: GBD 2019_Annex vitamin-A-deficiency.pdf [file pone.0266495.s004.pdf]

Vitamin A deficiency—Level 3 risk

Summary In 2019, vitamin A deficiency was responsible for 3·30 million (95% UI 1·35–5·59) DALYs and 23 800 deaths (2670–49 900), including 2·63 million (0·72–4·90) DALYs in children younger than 5 years.

Definition Vitamin A deficiency is defined as serum retinol less than 0·70 μmol/L and is a risk factor for diarrhoea and measles.

	Total sources
Exposure	274
Relative risk	16

Table 1: **Total sources used in GBD 2019 estimation**

What is new in GBD 2019?

- Vitamin A deficiency and vitamin A supplementation were modelled in ST-GPR to achieve improved time trends.
- Vitamin A supplementation estimates are now age-sex specific since supplementation campaigns target children.
- The age-specific stunting SEV was added as a covariate for vitamin A deficiency, alongside the three used last year: SDI, the availability of retinol activity equivalent (RAE) units in foods, and newly updated vitamin A supplementation.
- The evidence on vitamin A deficiency as a risk factor for diarrhoea, measles, and lower respiratory infections (LRIs) was re-analysed and evaluated using MR-BRT. LRIs were removed as an outcome due to insufficient evidence, and the relative risks for diarrhoea and measles were updated. Notably, we no longer adjust relative risks for background vitamin A deficiency prevalence.

	Deaths		YLLs		YLDs		DALYs	
	Number (millions)	Rate (per 100 000)	Number (millions)	Rate (per 100 000)	Number (millions)	Rate (per 100 000)	Number (millions)	Rate (per 100 000)
**2019**								
Both sexes	0·0238 (0·00267 to 0·0499)	0·4 (0·0 to 0·8)	2·08 (0·233 to 4·33)	31·3 (3·5 to 65·3)	1·22 (0·833 to 1·71)	17·6 (12·0 to 24·6)	3·30 (1·35 to 5·59)	48·9 (19·5 to 83·6)
Females	0·00987 (0·00102 to 0·0211)	0·3 (0·0 to 0·7)	0·859 (0·0892 to 1·83)	26·8 (2·8 to 57·0)	0·546 (0·373 to 0·768)	16·1 (11·0 to 22·8)	1·41 (0·593 to 2·42)	42·9 (17·6 to 74·5)
Males	0·0140 (0·00173 to 0·0289)	0·4 (0·1 to 0·8)	1·22 (0·151 to 2·51)	35·5 (4·4 to 73·2)	0·675 (0·459 to 0·943)	18·9 (12·9 to 26·4)	1·89 (0·763 to 3·23)	54·5 (21·7 to 93·5)
**Percentage change 2010–19**								
Both sexes	–60·1% (–68·0 to –50·1)	–60·5% (–68·3 to –50·5)	–60·1% (–67·9 to –50·1)	–60·5% (–68·3 to –50·5)	–22·1% (–26·1 to –18·4)	–25·7% (–29·4 to –22·3)	–51·3% (–59·8 to –32·2)	–52·5% (–60·6 to –34·8)
Females	–62·0% (–69·8 to –52·3)	–62·4% (–70·1 to –52·8)	–62·0% (–69·7 to –52·2)	–62·4% (–70·0 to –52·8)	–21·4% (–26·0 to –17·1)	–25·3% (–29·6 to –21·2)	–52·4% (–61·1 to –31·9)	–53·8% (–62·2 to –35·1)
Males	–58·6% (–67·1 to –47·3)	–59·0% (–67·4 to –47·7)	–58·6% (–67·1 to –47·3)	–59·0% (–67·4 to –47·7)	–22·7% (–27·8 to –17·5)	–26·0% (–30·9 to –21·1)	–50·4% (–59·3 to –31·5)	–51·5% (–60·2 to –34·0)

Numbers in parentheses are 95% uncertainty intervals.

Table 2: **Attributable global deaths, YLLs, YLDs, and DALYs in counts and age-standardised rates for both sexes combined, females, and males, 2019, with percentage change between 2010 and 2019**

	Deaths	YLLs	YLDs	DALYs
1990	36th	23rd	24th	22nd
2010	44th	40th	33rd	40th
2019	47th	44th	35th	47th

Table 3: **Rank among attributable Level 3 risks plus most detailed Level 2 risks of global deaths, YLLs, YLDs, and DALYs in 1990, 2010, and 2019 for both sexes combined**

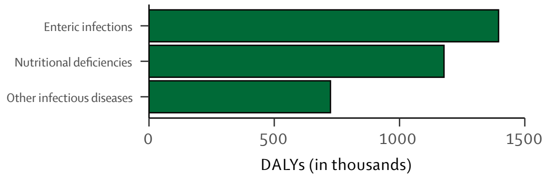

Figure 1: **Composition of attributable global DALYs by constituent Level 2 causes for both sexes combined, 2019**

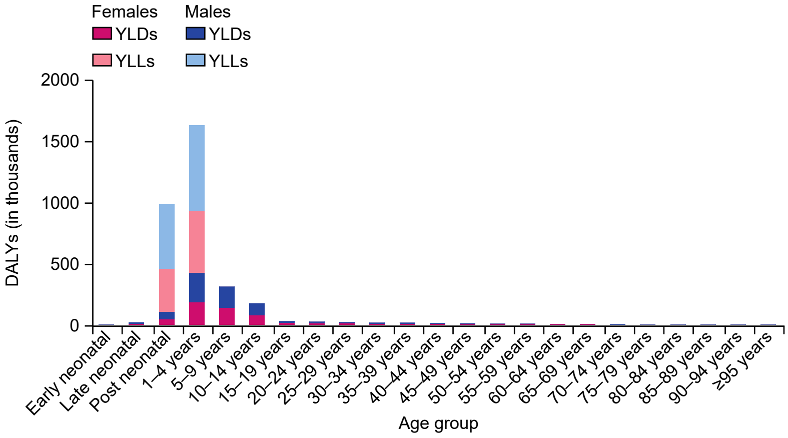

Figure 2: **Composition of attributable global DALYs by YLLs and YLDs, age group, and sex, 2019**

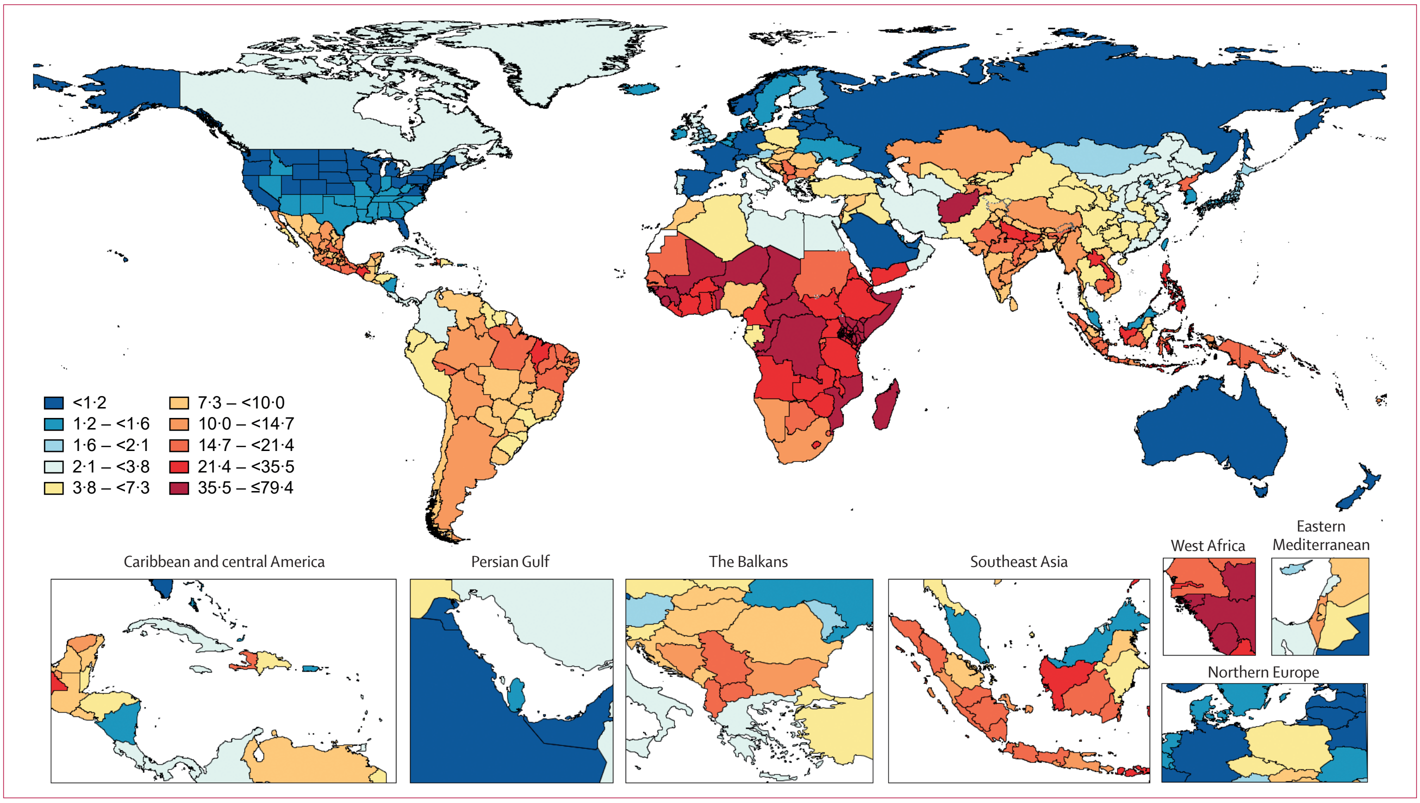

Figure 3: Age-standardised all-cause SEV by location, both sexes combined, 2019

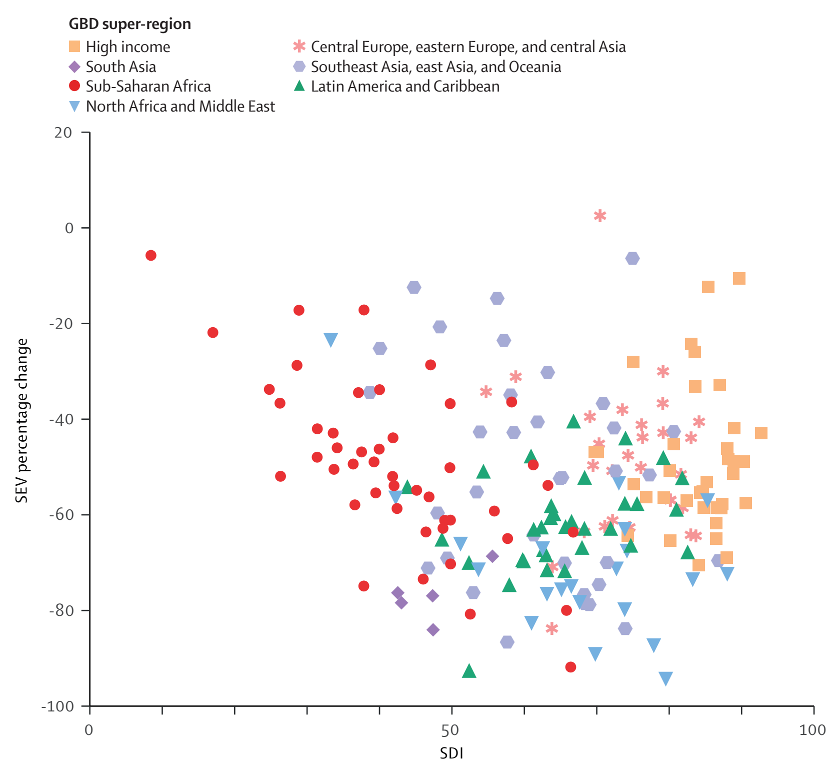

Figure 4: Percentage change in all-cause age-standardised SEV by SDI, both sexes combined, 1990–2019

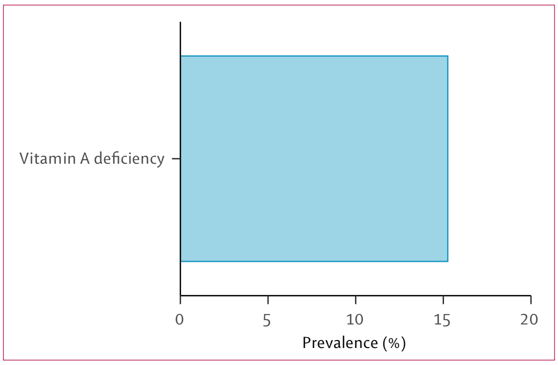

Figure 5: Percentage of population exposed to risk factor, both sexes combined, 2019

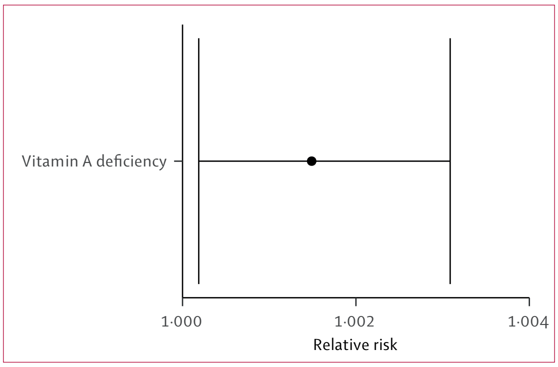

Figure 6: All-cause mortality relative risk, both sexes combined, 2019
