# Supplementary material for: Cost-effectiveness of Vitamin A supplementation among children in three sub-Saharan African countries: An individual-based simulation model using estimates from Global Burden of Disease 2019
Source: PLoS One. 2022 Apr 7;17(4):e0266495. doi: 10.1371/journal.pone.0266495 (PMC8989187; doi:10.1371/journal.pone.0266495)
Supplement: Attachment — Submitted filename: PLOS review_Dec20.docx [file pone.0266495.s003.docx]

The paper is very well thought out, very relevant, and timely in its topic. VAS is an essential child survival strategy in populations where VAD is a public health problem among children 6-59 mos of age. Despite it being very effective to reduce morbidity and mortality associated with VAD, it is an intervention that has been in place in some countries for over 20 years, and commitment to maintain such programs is waning in some countries. Cost-effectiveness data is critical to engaging policy makers, and advocating for continuation of this life-saving public health intervention especially as health systems are being stretched to the limits of their resources. For this reason we really welcome this analysis, and the rigour to which the authors brought to it.

Understanding that the authors simulated the scale up of VAS coverage from a hypothetical baseline of coverage to demonstrate that across three different settings, the effort to scale up the intervention is very cost effective.

I recommend however that this paper be revisited to consider the following:

1) The GBD 2019 significantly revised the methodology used and cited the following major changes that resulted in a very large reduction in # of DALYs due to VAD (reduced to 3.3M globally, and specifically 2.63M DALY’s in children U5):

- Vitamin A deficiency and vitamin A supplementation were modelled in ST-GPR to achieve improved time trends.
- Vitamin A supplementation estimates are now age-sex specific since supplementation campaigns target children.
- The age-specific stunting SEV was added as a covariate for vitamin A deficiency, alongside the three used last year: SDI, the availability of retinol activity equivalent (RAE) units in foods, and newly updated vitamin A supplementation.
- The evidence on vitamin A deficiency as a risk factor for diarrhoea, measles, and lower respiratory infections (LRIs) was reanalysed and evaluated using MR-BRT. LRIs were removed as an outcome due to insufficient evidence, and the relative risks for diarrhoea and measles were updated. Notably, we no longer adjust relative risks for background vitamin A deficiency prevalence.

Attached to this review is the Lancet ref, as well please see this recently published discussion paper: [Basis for changes in the global burden of disease estimates related to vitamin A and zinc deficiencies in the GBD 2017 and 2019 studies | Public Health Nutrition | Cambridge Core](https://www.cambridge.org/core/journals/public-health-nutrition/article/abs/basis-for-changes-in-the-global-burden-of-disease-estimates-related-to-vitamin-a-and-zinc-deficiencies-in-the-gbd-2017-and-2019-studies/0D3C57D1EF044BA7A1857E7D789F0134)

Could the authors consider re-running the analysis with the GBD 2019 data? If not, I strongly recommend this be acknowledged in the introduction as well as in the Discussion as a limitation, and consider what the ICER would be if the GBD 2019 was the source instead.

2) Line 67 references a paper that compared measles vaccination and therapeutic VAS in reducing mortality due to measles. While I am unable to access the full paper referenced (#10), it appears that that specific paper was considering the number of capsules given to treat a case of measles (in accordance with WHO Guidance on the therapeutic uses of VAS) – whereas the authors are comparing this to the two doses provided in PREVENTIVE programs. While preventive VAS can reduce incidence of measles by 50% (Imad et al 2017) – it is not found to reduce mortality due to measles.
3) Line 67, in addition to the above also has the following error: Large scale Food fortification does not reduce deaths associated with measles, LRI and diarrhoea – instead it is a strategy to increase the consumption of Vitamin A through the daily diet, and as such, can be considered as a means to reduce VAD in the population over time. Vitamin A supplementation on the other hand, only temporarily reduces VAD in the population, and so if a child consumes two high dose supplements, the supplement is protecting them from the effects of VAD (caused by the low intake of VA from the diet). As a result, VAS and fortification (and other dietary strategies) are complementary to each other – not alternatives. [The use and interpretation of serum retinol distributions in evaluating the public health impact of vitamin A programmes | Public Health Nutrition | Cambridge Core](https://www.cambridge.org/core/journals/public-health-nutrition/article/use-and-interpretation-of-serum-retinol-distributions-in-evaluating-the-public-health-impact-of-vitamin-a-programmes/EDDA029C9D15681577137087423D81FA)
4) Line 72 – refers to VAS as a treatment – however VAS programs are a preventive public health intervention. Recommend replacing “treatment” with the term “intervention”
5) Figure 1: this would change if GBD 2019 were considered instead
6) Line 128 – the cohort is referred to as children 0-59 months of age – however the eligible ages for VAS is 6 months to 59 months of age.
7) Line 139: VAS does not treat VAD. Recommend the change to “effectiveness of VAS to reduce VAD”
8) Line 161 that describes the source of the coverage data used in the simulation. Is there a reason why the authors did not consider using the globally available UNICEF database of national VAS coverage data by country? This data is administrative data, carefully reviewed and curated by UNICEF Data and Analytics, and publishes coverage by year, by country, by semester, and two-dose coverage –and can be found here: [Vitamin A Deficiency in Children - UNICEF DATA](https://data.unicef.org/topic/nutrition/vitamin-a-deficiency/) The user can also download the full excel dataset using the link on that page. This would be the better source for actual VAS coverage in a given country in a given year, as this is also the globally accepted source for VAS coverage data included in the Global Nutrition Report (GNR). If the authors do not decide to use the UNICEF data, I recommend the reason be included in the paper.
9) As a reviewer, my expertise is in the area of VAS programs, and Vitamin A nutrition and public health and I do not consider myself qualified to comment on statistical analysis, which is why I answered Q#2 in that manner.
10) Line 326: recommend the authors provide more context to make the connection to the Edejer paper and the relevance. In addition, suggest the authors also clarify if the $85/DALY result was all of the interventions combined, or each intervention at a time etc.
11) Line 360: could the authors build this out a little bit more to describe some additional cost drivers that are relevant to VAS programs. Costs are more likely to vary due to a) the delivery mechanism/or platform used in each country to deliver the VAS to children twice/year (routine, or campaign, or what kind of campaign), and b) the strength of the health system. Some countries need to spend a lot of money to deliver VAS to every child because the health system is not already reaching them. Did the authors feel there was sufficient data on costs in the literature? If not, could the authors make a recommendation of some kind – and comment on how this would improve the quality of future analysis such as this.
