# Supplementary material for: Cost-effectiveness of Vitamin A supplementation among children in three sub-Saharan African countries: An individual-based simulation model using estimates from Global Burden of Disease 2019
Source: PLoS One. 2022 Apr 7;17(4):e0266495. doi: 10.1371/journal.pone.0266495 (PMC8989187; doi:10.1371/journal.pone.0266495)
Supplement: Attachment — Submitted filename: PLOS ONE Review 12 21 21.docx [file pone.0266495.s005.docx]

Review of ‘Cost-effectiveness of Vitamin A Supplementation among Children in Three sub-Saharan African Countries: An Individual-based Simulation Model Using Estimates from the Global Burden of Disease 2017’

***Summary Assessment***: This is an interesting modeling exercise that has the potential to make useful contributions to the literature. However, substantial improvements are required to the current manuscript.

***Large Issues***:

1. The core results emerge logically from the assumptions and modeling approach – if more children receive VAS, there will be fewer VA-preventable deaths, and if program costs are linear then the cost-effectiveness parameters look promising. However, there are reasons to question the mortality-reducing impacts of VAS in today’s world (estimates are old and new ones cannot be generated for ethical reasons), and especially to question the linearity of costs of expanding coverage. There are reasons why coverage is so low in Nigeria, for example, and these suggest increasing costs as coverage increases, and perhaps some non-cost-related thresholds that need to be considered.
2. There is a big difference between inadequate intake of VA and VAD. A thorough analysis of the impact of a VAS campaign should consider both.
3. The authors use the suggestions that (e.g.) LSFF might be a better way of delivering VA to children to justify their analyses. However, no modeling is done to capture the cost-effectiveness of *alternative* VA deliver platforms, so the justification lacks punch. Vosti et al. (2020) and others have done such work.
4. It is not exactly clear what the authors mean when they say VAS; what are the VAS distribution details and associated cost functions? There are many ways to manage VAS programs, and the impacts and costs vary dramatically depending on the approach chosen.
5. Most countries in sub-Saharan Africa have found campaign-based VAS to be very expensive – indeed, if the international community were not covering the costs of these campaigns (including all inputs and many of the management costs), they would not be undertaken. So, what the authors suggests as unit costs seem not to jibe with reality; indeed, the country-level estimates of program expansion costs (even after adjusting for target population size) are an order of magnitude or more smaller than estimates offered by others. See Schott et al. 2021 for an example of the structure of costs for MNP distribution – not the same intervention, but the cost structure will be similar for VAS campaigns.
6. The authors note the importance of addressing uncertainty in their modeling efforts, but uncertainty regarding the impact of VAS on VAD-attributable mortality and (especially) regarding scale-up costs seem not to have received such attention.
7. The authors contend that VAS is a highly cost-effective intervention; new literature and ongoing experience in the field suggest that this may not be the case. This is an especially contentious claim based on linear costs of scaling up VAS programs.

***Smaller Issues***:

1. It is not clear why these three countries were chosen.
2. The authors admit that there are no YLD parameters in this simulation exercise, so best to exclude them. Indeed, I would suggest presenting results in terms of deaths averted (rather than DALYs), since no comparisons are being made with other interventions with different mortality/disability structure.
3. It is not clear what ‘individual-based’ means, nor it is clear what calibrations were undertaken to ‘match’ (how well?) GBD estimates. What are SIR and SIS models? What does fixed-effect metanalysis mean, in the several places in which the term is used? Generally, the paper is not accessible to those outside the IHME and their sphere of influence; this can be fixed, but some additional effort will be required.
