# Supplementary material for: Cost-effectiveness of Vitamin A supplementation among children in three sub-Saharan African countries: An individual-based simulation model using estimates from Global Burden of Disease 2019
Source: PLoS One. 2022 Apr 7;17(4):e0266495. doi: 10.1371/journal.pone.0266495 (PMC8989187; doi:10.1371/journal.pone.0266495)
Supplement: Attachment — Submitted filename: Response to Reviewers.docx [file pone.0266495.s007.docx]

# Response to reviewers for Vitamin A Supplementation

## Reviewer 1

*One outstanding question for me: The VAD prevalence data indicated in Table 1 seems off by quite a bit - for instance WHO estimates for VAD among PRESAC children in Nigeria is in the range of 15-25% -- whereas Table 1 states it as 1.2%. What is the data source for the VAD prevalence used in the analysis? GBD 2019 also indicates the analysis no longer corrects for background VAD - whereas the explanation of the difference in results is partially due to the differences in VAD prevalence. Please review before proceeding.*

We thank the reviewer for pointing out this issue in Table 1. We reviewed this issue and found that indeed the prevalence for VAD in Table 1 deviated from previous estimates.

These values were generated from the GHDx GBD Results tool. We found that the values for VAD prevalence were not updated properly in GHDx for GBD 2019 due to a last-minute resubmission. We updated Table 1 to accurately reflect the inputs for VAD prevalence from GBD 2019. We also added prevalence values for Measles and Diarrheal Diseases that were inputs in the simulation.

Fortunately, the values for VAD risk exposure that we used for the simulation were updated already to include the correct GBD estimates, so this change does not affect the outputs of the simulation.

## Reviewer 2

*The updated manuscript is much improved; indeed, it is much clearer and the conclusions have been completely reversed! There are still some additional work that could be done (e.g., additional sensitivity analysis regarding some key model parameters and assumptions), but to my mind, the authors have met the criteria for publication. Congrats to all!*

Thank you very much for this positive assessment of our work.
